# Supplementary material for: Comparison of five different disseminated intravascular coagulation criteria in predicting mortality in patients with sepsis
Source: PLoS One. 2024 Mar 7;19(3):e0295050. doi: 10.1371/journal.pone.0295050 (PMC10919643; doi:10.1371/journal.pone.0295050)
Supplement: Attachment — Submitted filename: Response to Reviewers.docx [file pone.0295050.s002.docx]

To
Yoshihisa Tsuji
Academic Editor
PLOS ONE

Subject: Response to reviewers’ comment on Manuscript titled “Comparison of five different Disseminated Intravascular Coagulation Criteria in predicting mortality in Patients with Sepsis” with ID # “PONE-D-23-14306”

Dear Editor

We are grateful for the comments of the esteemed reviewers and have formulated a rebuttal letter (attached below) for their kind review. The rebuttal letter contains the details of the changes made to the manuscript in line with the reviewers’ comments. Moreover, the changes have been marked as track changes in the manuscript file. The page and line number in the rebuttal letter can be matched with the tracked changes file. In addition, we have included the SPSS data file to support our findings.

We are hopeful that our responses will satisfy the reviewers’ panel and will get a favorable reply from the Journal

Thanks, and Regards

Dr. Saad Bin Zafar Mahmood (Corresponding Author)
Senior Instructor and Consultant Physician
Aga Khan University Hospital
Karachi – Pakistan
[Saadbin.zafar@aku.edu](mailto:Saadbin.zafar@aku.edu)

Reviewer 1: The Manuscript compared the performance of five DIC diagnostic criteria focusing on the prediction of mortality in Pakistan. A retrospective cohort study using electronic medical records was conducted. The authors concluded that Japanese Association for Acute Medicine (JAAM) and Korean Society on Thrombosis and Hemostasis (KSTH) criteria were most valuable diagnostic criteria for screening septic patients for DIC in Pakistan population.

| S. No | Comment | Response | Page/Line Number |
| --- | --- | --- | --- |
| 1 | The details of what sepsis is in ICD-9 was not written specifically. A description of what sepsis is included is needed. | We acknowledge this shortcoming. Dombrovskiy definition of sepsis was taken and has now been explained in methodology. The new reference has also been included (#11) | Page 6 Line 111-115 |
| 2 | There is no data description regarding the implementation of DIC score assessment. Basically, DIC scores do not use the parameters from time points apart in time. Are the parameters measured at the same time? What percentage of the evaluation was done on day 1？ | We acknowledge that this data is missing. This has now been included  “All the elements used to assess DIC criteria for each patient originated from a single day. On the initial day, 44.3% underwent DIC evaluation based on clinical judgment, while on the second day, the assessment was conducted for 33.3%. On the third day, 13.1% underwent evaluation, followed by 4.2% on the fourth day, and 3.4% on the fifth day. The remaining four patients underwent DIC evaluation on days 6, 8, 10, and 11, respectively.” | Page 7, Line 139-143 |
| 3 | A significant confounding factor regarding DIC diagnosis and mortality is intervention for DIC. The information about ICU admission rates and treatment for DIC needed. | We acknowledge this deficiency. The data for ICU admission rates and outcome was available and has been included. 89 patients (40.1%) were intubated and experienced an Intensive Care Unit stay.  However, treatment received has not been included and has been acknowledged as a limitation. | Page 11, Line 184  Page 19, Line 318-321 |
| 4 | It is not mentioned whether the DIC score evaluator was aware of the information regarding patient outcomes. | Since this is a retrospective study, medical record files were systematically reviewed and patients meeting inclusion criteria were included. Scores were applied as per the criteria and outcome was noted from the records by the same data collector. This detail has been included in the methodology also. | Page 6, Line 124-127 |
| 5 | All of 5 criteria seem to have low predictive accuracy. Nevertheless, the authors recommend the use of the DIC score, but what is the advantage of recommending its use over other mortality predictors? I think the conclusion should be changed. | We have changed the conclusion to state  Our study findings indicate that among the five evaluated models of DIC, KSTH demonstrated superior sensitivity and accuracy, while R-JAAM exhibited the highest specificity and positive predictive value for mortality prediction in sepsis patients as compared to others. Our study results highlight the need for large scale studies to validate these findings before generalizing it and making it a part of local guidelines. | Page 20, Line 336-341 |
| 6 | For external validation, information on the clinical setting is needed. For example, information on patient transport routes and ICU admission rates is needed. | We have added more information regarding the clinical setting.  Aga Khan University Hospital is one of the largest academic tertiary care centers in South Asia. It is a 600 bed facility with a state of the art emergency department and rooms of different acuities; low-monitoring ward beds with a nurse ratio of approximately 5:1, high dependency units with 24 hour cardiac monitoring, non-invasive mechanical ventilation facilities and nurse ratio of 5:2, and intensive care units (ICU) with mechanical ventilator facilities and nurse ratio of 1:1. In addition, the hospital has a rapid response team consisting of an ICU nurse and doctor and is the first responder to hospital areas in case of any emergency. The hospital also has a robust transport mechanism for patients who need to be shifted to ICU. Such patients are accompanied by an Advanced cardiac life support (ACLS) certified nurse and doctor along with the RRT nurse. | Page 5, Line 100-109 |
| 7 | No unit description for lactate in Line 168. | The unit for lactate (mM/L) has been added | Page 11, Line 189-190 |

Reviewer 2: This study compared the 5 different DIC criteria and revealed the diagnostic accuracy for septic DIC as well as prediction of mortality. Unfortunately, I cannot accept this study for the following reasons. Please see comments below.

| S. No | Comment | Response | Page/Line Number |
| --- | --- | --- | --- |
| 1 | Internal validity In this study, the relationship between DIC and patient outcomes were analyzed, but never analyzed some important confounding factors such as comorbidities. | We agree that data on comorbidities is necessary. The two most important comorbidities diabetes and hypertension have now been included in Table 2 | Page 12, Line 213 |
| 2 | External validity It is difficult to apply the results of the 222 patients included in the study to patients with sepsis in general. The number included in the study is too small to generalize the result. | We have changed the conclusion to state  Our study findings indicate that among the five evaluated models of DIC, KSTH demonstrated superior sensitivity and accuracy, while R-JAAM exhibited the highest specificity and positive predictive value for mortality prediction in sepsis patients as compared to others. Our study results highlight the need for large scale studies to validate these findings before generalizing it and making it a part of local guidelines. | Page 20, Line 336-341 |
| 3 | Feasibility The authors recommend the combined use of JAAM and KSTH criteria for septic DIC. This means “ double standard “ in the diagnosis, which is inconsistent with current practice of sepsis. | We have changed the conclusion to state  Our study findings indicate that among the five evaluated models of DIC, KSTH demonstrated superior sensitivity and accuracy, while R-JAAM exhibited the highest specificity and positive predictive value for mortality prediction in sepsis patients as compared to others. Our study results highlight the need for large scale studies to validate these findings before generalizing it and making it a part of local guidelines. | Page 20, Line 336-341 |
| 4 | Novelty The authors mentioned Reference No.8 evaluating 5 DIC criteria like them, and the results were different between them. They never explained the reason of the inconsistent results. The novelty was unclear. | We have explained the reason with a source reference  Overall, comparison between two studies would be difficult as one was prospective while the other was retrospective. Moreover, it is important to note that each population has different racial characteristics and hence different thrombolytic mechanisms. | Page 17, Line 264-267 |
