# Supplementary material for: Comparison of five different disseminated intravascular coagulation criteria in predicting mortality in patients with sepsis
Source: PLoS One. 2024 Mar 7;19(3):e0295050. doi: 10.1371/journal.pone.0295050 (PMC10919643; doi:10.1371/journal.pone.0295050)
Supplement: Attachment — Submitted filename: Response to Reviewers_2_191023.docx [file pone.0295050.s003.docx]

To
Yoshihisa Tsuji
Academic Editor
PLOS ONE

Subject: Response to reviewers’ comment on Manuscript titled “Comparison of five different Disseminated Intravascular Coagulation Criteria in predicting mortality in Patients with Sepsis” with ID # “PONE-D-23-14306R1”

Dear Editor

We are grateful for the comments of the esteemed reviewers and have formulated a rebuttal letter (attached below) for their kind review. The rebuttal letter contains the details of the changes made to the manuscript in line with the reviewers’ comments. Moreover, the changes have been marked as track changes in the manuscript file. The page and line number in the rebuttal letter can be matched with the tracked changes file.

We are hopeful that our responses will satisfy the reviewers’ panel and will get a favorable reply from the Journal

Thanks, and Regards

Dr. Saad Bin Zafar Mahmood (Corresponding Author)
Senior Instructor and Consultant Physician
Aga Khan University Hospital
Karachi – Pakistan
[Saadbin.zafar@aku.edu](mailto:Saadbin.zafar@aku.edu)

Reviewer 1:

| S. No | Comment | Response | Page/Line Number |
| --- | --- | --- | --- |
| 1 | Thank you for providing more information on the ICD-9 criteria for inclusion based on Dombrovskiy's work, but reading Dombrovskiy's article, it seems to cover severe sepsis based on sepsis-1. Is it correct to understand that this corresponds to "sepsis" according to today's sepsis-3 definition? A definition of sepsis is needed. | Thank you for taking the time to review our article, and we appreciate your feedback. Your attention to detail is invaluable in helping us improve the clarity of our work.  As required the definition of sepsis has been added as mentioned below with the appropriate reference.  “Sepsis was defined as life-threatening organ dysfunction caused by a dysregulated host response to infection”  However, global literature itself acknowledges the inconsistent strategies in selecting ICD codes for sepsis and the resulting problem. Most of these ICD codes for sepsis have been categorized before 2016 when Sepsis-3 definition was not in use.  The first JAMA paper (Singer M, et al. The Third International Consensus Definitions for Sepsis and Septic Shock (Sepsis-3). JAMA. 2016 Feb 23;315(8):801-10.) on Sepsis-3 defined sepsis as per the definition mentioned above and advised to use only two explicit codes (995.92 and 785.52) for sepsis categorization.  However, a most recent article by Rudd and colleagues (Rudd et al. Global, regional, and national sepsis incidence and mortality, 1990–2017: analysis for the Global Burden of Disease Study. Lancet. 2020; 395: 200-211) and an extended commentary by Duke and colleagues accepts that using only explicit codes mentioned above may be inconsistent, unreliable and underestimate the true prevalence of sepsis.  The advice by these authors is that sepsis diagnosis should require two simultaneous diagnosis codes: one for an infectious disease and another for an acute organ dysfunction diagnosis. Unfortunately, the new categorization being done now is based on ICD-10-CM codes, whereas our hospital has switched to ICD-10-CM codes from 2022 and all previous coding has been done based on ICD-9-CM codes. Therefore, we utilized the existing work of Dombrovskiy's for ICD-9-CM code list from the work of Rhee and others, for the Centers for Disease Control and Prevention Epicenters Program as mentioned in the manuscript. This ICD-9-CM coding was also approved and provided by our Hospital Management Information System (HIMS) office tasked with maintaining all the patient data and categorizing them according to ICD codes.  I would like to clarify that the ICD-9-CM codes in this article mentioned under the heading of Dombrovskiy's definition do enroll severe sepsis patients but based on the same definition of having a source of infection along with an evidence of organ dysfunction. This definition is comparable to sepsis definition as per sepsis-3 which is applicable nowadays as also seen in the JAMA paper quoted above.  However, to enhance the validity of our study, we also included the modified Martin Criteria as done by Bouza and colleagues, Fortunately, there were only a few additional ICD-9-CM codes that were added as mentioned in text  “and Modified Martin Criteria (Infection codes: 038.0 - 038.4, 038.8, 038.9, 003.1, 020.2 , 036.2, 036.3, 054.5, 098.89, 112.5, 112.81, 117.9, 790.7, 995.91 AND organ dysfunction codes: 518.81, 518.82, 518.84, 518.85, 786.09, 799.1, 785.5 with all sub codes, 458 , 796.3 , 584 with all sub codes, 580 , 570, 572.2, 573.3, 286.6, 286.9, 287.3-5, 293, 348.1, 348.3, 357.82, 780.01, 780.09, 276.2)”  However, importantly no additional entries were obtained in the study cohort likely because the coding done by the HIMS office is based on the Dombrovskiy's code list.  To further add, we have added the ICD coding issues as a limitation in our manuscript  “Moreover, ICD-9-CM coding for sepsis has been found to be categorized differently across literature and needs to be looked thoroughly between different institutions.”  I believe that this explanation would help in clarifying any remaining issues regarding the sepsis definition in our manuscript. Moreover, the study’s main objective is to compare the different DIC criteria, and we believe that the study methodology can easily be replicated at another institution using ICD-9-CM or ICD-10-CM codes as per the hospital ICD code list. | Page 6, Line 109-121  Page 10, Line 321-322 |
| 2 | According to the #11 literature, the classification is by ICD-9 CM Code, not ICD-9.  Which coding is correct? | We apologize for this misconception.  The ICD-9 is just an abbreviated form of the full ICD-9-CM Code. ICD-9-CM is the official system of assigning codes to diagnoses and procedures associated with hospital utilization. This is abbreviated to ICD-9.  To make it easy and clear for the readers we have used the term “ICD-9-CM” all over the manuscript now. |  |
| 3 | Thank you for describing the data on the implementation of the DIC score assessment. I understand that the evaluations are carried out at different points in time, does this mean that the worst values are used as data? Do you mean the initial assessment? Please describe it in a way that the reader can understand. | We appreciate your diligence in seeking clarification.  The following text is already included in the methodology section but has been highlighted again in the manuscript.  “All the elements used to assess DIC criteria for each patient originated from a single day. On the initial day, 44.3% underwent DIC evaluation based on clinical judgment, while on the second day, the assessment was conducted for 33.3%. On the third day, 13.1% underwent evaluation, followed by 4.2% on the fourth day, and 3.4% on the fifth day. The remaining four patients underwent DIC evaluation on days 6, 8, 10, and 11, respectively. Hence, these investigations were carried out on the day when the clinician suspected the patient might be experiencing DIC. These values are not the worst reported values during the admission but were done on a single day” | Page 7, Line 144-151 |
| 4 | Regarding results. I calculated the performance of the JMHW from the data in the manuscript, but there is concern that the results for specificity, positive predictive value and accuracy are incorrect. My calculations yielded a specificity of 61.8%, a PPV of 78.0% and an accuracy of 60.3%.  If the results of the calculations are in error, the performance difference in the five criteria appears to be narrowing.  If the results change, the conclusions should be re-considered. | We are extremely thankful for highlighting the numerical inaccuracies within Table 4. These errors have been thoroughly reviewed and rectified in the manuscript. We can confirm that all other numerical data has been meticulously scrutinized and found to be accurate.  Fortunately, these corrections do not alter the overall conclusions of the manuscript, as our study does not offer a definitive criterion based on our findings, rather encourage the researchers to consider replicating this study in a prospective manner to yield more robust results considering the high overall mortality. However, the required value changes have been made in the table and text. |  |
| 5 | It should be stated in the Limitation that the timing of coagulation testing is dependent on clinician bias and that the therapeutic interventions for the diagnosis of DIC are unknown. | We do agree with your opinion on both accounts and have made the changes. This was already mentioned in the manuscript, but we have further clarified it based on your valuable suggestion.  The following text has been added in limitations to further clarify the limitations.  “The selection of patients who underwent coagulation testing relied on the clinical judgment of physicians, representing a notable constraint in our study.”  Regarding therapeutic interventions the following lines were already present.  “Moreover, our study also did not include the treatment regimens for these patients due to the variability and since there is yet to be an international consensus on whether DIC should be a therapeutic target with anticoagulant therapy” | Page 20, Line 324-330 |

Reviewer 2: The authors answered all questions adequately and reflected them in the text. I have no further comments.

Thank you for taking the time to review our article, and we appreciate your feedback.
